# Friend Ahoy!

**Published:** 1874-03

**Authors:** 


### FRIEND AHOY!

"As ships meet at sea, a moment together, when words of greeting must be spoken, and then away into the deep, so men meet in this world; and I think we should cross no man's path without hailing him, and, if he needs, giving him supplies."—*Henry Ward Beecher.*

Friend ahoy! How many days
Hast thou been out? How many nights
Did friends stand watching on thy ways?
Do lovers trim the lights?

Friend ahoy! Art thou in need
Of aught we carry? Make but sign
Which we across the waves may read,
And all our store is thine.

Friend ahoy! Draw near—draw near!
Let us, at least one short hour, sail
Close side by side. Let words of cheer
Over our griefs prevail.

Friend ahoy! The waves toss white;
Rises the wind which parts us far;
We shall ride out the stormy night
By help of the selfsame star.

Friend ahoy! Farewell—farewell!
Grief unto grief, joy unto joy,
Greeting and help the echoes tell,
Faint but eternal—Friend ahoy!

—*Christian Union.*

